# Combining Mechanochemistry and Spray Congealing for New Praziquantel Pediatric Formulations in Schistosomiasis Treatment

**DOI:** 10.3390/ijms20051233

**Published:** 2019-03-12

**Authors:** Beatrice Albertini, Beatrice Perissutti, Serena Bertoni, Debora Zanolla, Erica Franceschinis, Dario Voinovich, Flavio Lombardo, Jennifer Keiser, Nadia Passerini

**Affiliations:** 1 Department of Pharmacy and BioTechnology, University of Bologna, Via S. Donato 19/2, 40127 Bologna, Italy; serena.bertoni4@unibo.it; 2 Department of Chemical and Pharmaceutical Sciences, University of Trieste, P.le Europa 1, 34127 Trieste, Italy; bperissutti@units.it (B.P.); debora.zanolla@gmail.com (D.Z.); vojnovic@units.it (D.V.); 3 Department of Pharmaceutical and Pharmacological Sciences, University of Padova, via Marzolo 5, 35131 Padova, Italy; erica.franceschinis@unipd.it; 4 Helminth Drug Development Unit, Department of Medical Parasitology and Infection Biology, Swiss Tropical and Public Health Institute, Socinstr.57, CH-4051 Basel, Switzerland; flavio.lombardo@unibas.ch (F.L.); jennifer.keiser@swisstph.ch (J.K.); 5 Universität Basel, Petersplatz 1, P.O. Box, CH-4001 Basel, Switzerland

**Keywords:** poorly water soluble drug, solubility enhancement, grinding, spray congealing, neglected tropical diseases, polymorph

## Abstract

Praziquantel (PZQ) is the first line drug for the treatment of schistosome infections and is included in the WHO Model List of Essential Medicines for Children. In this study, the association of mechanochemical activation (MA) and the spray congealing (SC) technology was evaluated for developing a child-friendly PZQ dosage form, with better product handling and biopharmaceutical properties, compared to MA materials. A 1:1 by wt PZQ—Povidone coground—was prepared in a vibrational mill under cryogenic conditions, for favoring amorphization. PZQ was neat ground to obtain its polymorphic form (Form B), which has an improved solubility and bioactivity. Then, activated PZQ powders were loaded into microparticles (MPs) by the SC technology, using the self-emulsifying agent Gelucire^®^ 50/13 as a carrier. Both, the activated powders and the corresponding loaded MPs were characterized for morphology, wettability, solubility, dissolution behavior, drug content, and drug solid state (Hot Stage Microscopy (HSM), Differential Scanning Calorimetry (DSC), X-Ray Powder Diffraction Studies (PXRD), and FT-IR). Samples were also in vitro tested for a comparison with PZQ against *Schistosoma mansoni* newly transformed schistosomula (NTS) and adults. MPs containing both MA systems showed a further increase of biopharmaceutical properties, compared to the milled powders, while maintaining PZQ bioactivity. MPs containing PZQ Form B represented the most promising product for designing a new PZQ formulation.

## 1. Introduction

Schistosomiasis is one of today’s foremost neglected tropical diseases (NTDs) and a disease of poverty affecting more than 200 million people, worldwide [1]. NTDs are a group of 17 endemic diseases that prevail in less developed areas where large numbers of people have little or no access to adequate health care, clean water, housing, transport, and information [2]. In particular, Schistosomiasis is a tropical and subtropical disease caused by one of the six different species of the trematode worm *Schistosoma*, with the great majority of cases either infected with *Schistosoma haematobium*, *S. japonicum* or *S. mansoni* [3,4]. In the absence of effective vaccines for helminthic infections, praziquantel (PZQ) is the first line drug used in endemic countries, for the treatment and prevention therapy of schistosome infections [1,5], and is included in the WHO Model List of Essential Medicines for Children [6]. However, there is still a need for discovering alternative treatments and superior formulations [1], especially for pre-school-age children and infants, which require more appropriate dosage forms, in terms of ease of dose adjustment and swallowing [7]. In fact, PZQ is available as a 600 mg film-coated tablet, and a high dose (maximum 40 mg/kg bodyweight) is required. More recently, 150 mg tablets have become commercially available [8].

From the biopharmaceutical point of view, several approaches have been undertaken to reduce the PZQ therapeutic dose, due to its solubility enhancement [7,9,10]. Recently we have proposed some strategies involving the mechanochemical activation of the drug, proving the amelioration of the PZQ biopharmaceutical properties (solubility, intrinsic dissolution rate (IDR)) [11,12,13]. In particular, in the first study, PZQ was coground with different pharmaceutical polymers, using a lab-scale vibrational mill and an explorative analysis of formulation variables (drug-polymer wt. ratio and polymer type), and process-related parameters (type of grinding media, grinding time, and frequency) was carried out with the help of an experimental screening design. The most promising sample, in terms of drug solubility enhancement, was a coground composite with crospovidone, with a 50%-by-weight drug content, permitting a 4.6-fold solubility improvement, in comparison to the starting drug. This product displayed a high amorphous character, as it was able to maintain the in vitro bioactivity of the PZQ, against the *S. mansoni*, and appeared to be chemically and physically stable, over six ageing months [11]. Considering that this system revealed an incomplete drug recovery (88.01%), a second research experience encompassed the use of the cryomilling technique (using liquid nitrogen as a cryogenic media), to prevent chemical degradation of the drug during comilling, and to improve activation efficiency. The most promising polymers selected from the former experience (linear and cross-linked povidone) were then used to successfully prepare cryo-composites with a high PZQ content. Cryomilling PZQ with linear povidone led to a significant decrease of drug degradation (PZQ recovery ranged from 95.2% to 98.9%), along with a remarkable reduction of the crystalline content (ranging from 47% to 0%). From both experiences it was clear that the drug, when comilled in presence of polymers, was more prone to amorphization and a certain amount of degradation. Conversely, PZQ that was ground by itself, did not show this propensity to degradation while maintaining the tendency to physical transformations [13]. In addition, when subjected to neat grinding in adequate conditions, PZQ evidenced to form an anhydrous polymorphic variety with a very high efficiency. The obtained new polymorph, named Form B, displayed a two-fold increased water solubility and IDR, in comparison to PZQ, and a better activity than the raw PZQ [12]. Therefore, all these attempts testified to the possibility of forming different activated crystal modifications of PZQ (amorphous/nanocrystalline or polymorphous) with ameliorated biopharmaceutical properties, by means of a mechanochemical process.

With the final ambitious aim of developing a child-friendly PZQ dosage form, a further solubility improvement and a better product handling with respect to the milled systems (drug and coground mixture), was then pursued. In the development of pediatric formulation, an important concern is the appropriate choice of excipients. The EMA “Guideline on pharmaceutical development of medicines for pediatric use” suggests to avoid any excipients that are potentially toxic or unsuitable for children [14]. In this study, the association of the mechanochemical activation with the formation of a PZQ solid dispersion by the spray congealing technology was evaluated. Raw PZQ, its physical mixture with polyvinylpyrrolidone (50:50 *w*/*w*) and activated materials, such as the new polymorphic form, Form B, and the cryo-coground of PZQ:PVP (50:50 *w*/*w*) were, thus, loaded into spray congealed microparticles. The 1:1 drug to polymer wt. ratio was selected, since it showed a significant improvement in solubility performance than a 1:2 ratio [11]. As microparticle carrier, Gelucire^®^ 50/13 was selected, since it formerly demonstrated its great ability to improve the biopharmaceutical properties of several active ingredients—silybum Marianum dry extract [15], glibenclamide [16], carbamazepine [17], melatonin [18], and piroxicam [19]. No safety concerns regarding the selected excipients (PVP and Gelucire^®^ 50/13) were documented. 

Both, the activated materials and the corresponding loaded microparticles were then fully characterized with regards to morphology (SEM and particle size), wettability, solubility, dissolution behavior, drug content, and drug solid state (Hot Stage Microscopy (HSM), Differential Scanning Calorimetry (DSC), X-Ray Powder Diffraction Studies (PXRD), and FT-IR). The results were then compared to those obtained from the raw materials (PZQ and physical mixture). Finally, the loaded microparticles were tested, in vitro, in comparison to PZQ, against the *S. mansoni* newly transformed schistosomula (NTS) and adults.

## 2. Results and Discussion

### 2.1. Analysis of the Activated Materials

The characteristics of the activated materials were analyzed, prior to their addition to the microparticles. Table 1 summarizes the results of the HPLC assay and of the thermal analysis. Regarding the HPLC analysis, all impurities were absent, both in the standard solution (raw PZQ) and in Form B, prepared by neat grinding, indicating that milling process of the pure drug induced a physical transformation but not the formation of impurities. Thus, the PZQ recovery was 100%. In the case of cryo-coground sample (obtained by vibrating for 60 min at 20 Hz), the PZQ recovery slightly decreased and the total amount of impurity was about 1.11%. Further, analogous to our previous recent study [12], the presence of an additional compound, indicated as “X” impurity, which has not been reported in the Ph-. Eur. PZQ monography or in that of the USP, was detected. This compound, having a molecular ion m/z 147, was identified in a previous research work [20]. Impurity X always showed a higher concentration than impurity A, which never exceeded the 0.2% (the acceptance criteria of the USP 36-NF 31). Conversely, impurity B was never detected. All ground samples were, hence, in accordance with the E.P. criteria. This result displayed that the milling process realized in the suitable conditions did not induce a significant chemical degradation of PZQ; therefore, both of the activated materials were considered suitable for their loading into microparticles.

As previously stated, one of the main aims of the milling procedure for a poorly bioavailable drug, is its solubility enhancement. The results of the solubility test, reported in Table 1, clearly showed a 2-fold improvement of the solubility, in comparison to raw PZQ. In particular, both the cryo-comilled sample and Form B was found to be significantly more soluble than raw PZQ and the physical mixture, although no significant difference between the two activated samples was found.

**Figure 1:**
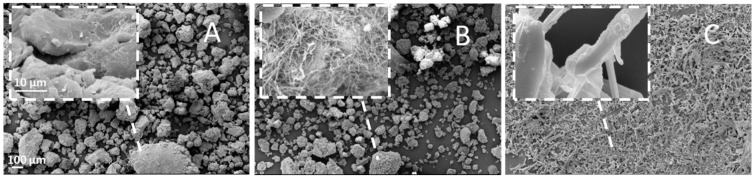
SEM images of PZQ:PVP CC (**A**), PZQ Form B (**B**), and raw PZQ (**C**) (magnification 150 x; in the top left frame a 5.0 K× magnified image).

SEM pictures, presented in Figure 1, show that starting PZQ had an acicular habitus (Figure 1C), while Form B had a very different habit, consisting of agglomerates of long and very thin whiskers (Figure 1B). In the CC sample (Figure 1A), compared to primary particles, the particles exhibited a significant change in the shape and surface morphology, all showing irregular blocky-shaped particles. In fact, the PVP particles their lost typical spherical shape and drug crystal were no longer recognizable during cryo-cogrinding. These phenomena suggest that the raw materials were intimately and homogenously combined in the CC, and PZQ particles were completely covered by the PVP macromolecules.

Laser light scattering analysis revealed that the original PZQ had a mono-modal particle size distribution with a median diameter of 23.90 µm. PZQ Form B had a d_(0.1)_ of 20.90 µm, d_(0.5)_ of 77.44 µm, and a d_(0.9)_ 139.14 µm. PVP particles showed the following size distribution d_(0.1)_ of 25.50 µm, d_(0.5)_ of 76.20 µm, and a d_(0.9)_ 130.78 µm. When the PZQ was physically mixed with PVP in a 1:1 wt. proportion, a multimodal size distribution was obtained, with peaks in correspondence to those of the components. The CC system showed a variation of the particle size, with respect to the untreated mixture of powders, reaching a median diameter of 243.99 µm, in agreement with the SEM pictures reported in Figure 1.

**Figure 2:**
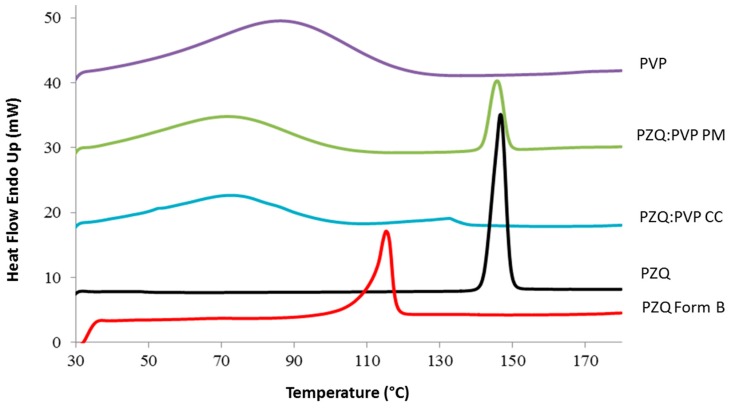
Differential Scanning Calorimetry (DSC) curve overlay of raw PZQ, PVP K30, PZQ:PVP PM, and activated materials (PZQ:PVP CC and PZQ Form B).

Then, the thermal analysis was conducted to check the solid state transformation of PZQ, after the milling process. Table 1 reports the temperatures of the PZQ endotherm and the calculated residual crystallinity of the analyzed samples; while Figure 2 shows the corresponding DSC curves. The DSC scan of PZQ showed only a single endothermic peak at about 143.5 °C (∆H= 91.9 ± 5.32 J/g), in agreement with the melting point and enthalpy of fusion of the racemic form of the drug [7]. In the physical mixture, this event was anticipated by the dehydration of the hygroscopic polymer, as PVP K30 showed only a large dehydration peak between 50 and 100 °C. The CC sample evidenced a lowering of the PZQ thermal event, due to the intense physical disruption of the crystalline structure—the endothermic peak was lower than the original racemic PZQ (see Table 1), in agreement with a very scarce residual crystallinity and with the prevalent presence of the nanocrystals of PZQ. In fact, the lower the nanocrystal size, the lower was its melting temperature and enthalpy [21]. In the case of the PZQ Form B, the endothermal event corresponding to its melting point appeared at about 112.1 °C (∆H= 55.9 ± 1.5 J/g), according to Zanolla et al. [12].

**Figure 3:**
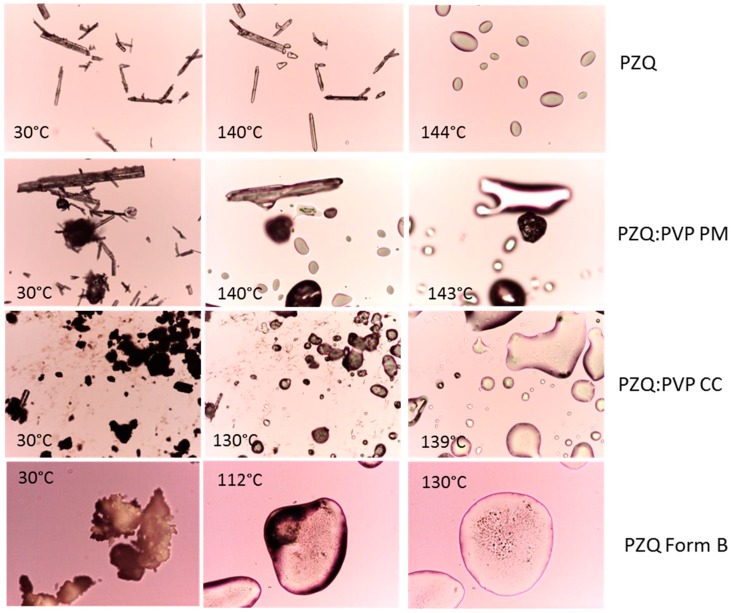
Hot Stage Microscopy (HSM) analysis of raw PZQ, PVP K30, physical mixture, and activated materials (magnification 10×).

Hot stage microscopy analysis clearly showed physical changes in the samples, during the temperature scan (Figure 3). PZQ showed elongated acicular crystals of different sizes, which completed their fusion at 144 °C. A slight PZQ melting point reduction to 140 °C was visible by analyzing the physical mixture (due to the dilution effect). The HSM pictures of the PZQ:PVP CC and PZQ Form B confirm the fusion at about 130 °C and 112 °C, respectively. 

**Figure 4:**
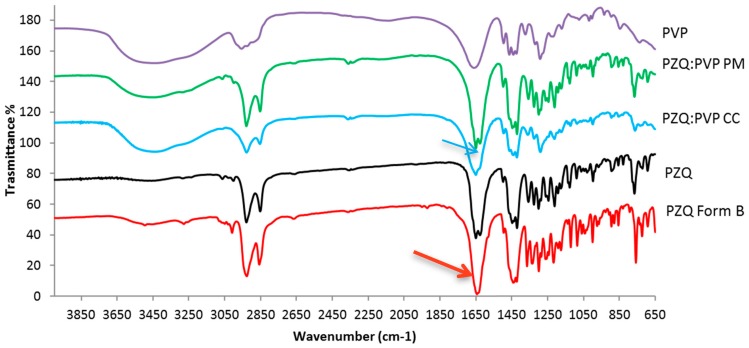
FT-IR spectra of raw PZQ, PVP K30, PZQ:PVP PM and activated materials (PZQ:PVP CC and PZQ Form B). The arrows show the carbonyl stretching vibration.

FTIR was then performed to detect structural modifications and possible interactions among the components in milled samples (Figure 4). The FTIR spectrum of PZQ shows characteristic peaks at 2929 cm^−1^ and 2852 cm^−1^, due to the C–H and C–H_2_ stretching vibration and an intense multiband at 1650–1600 cm^−1^ due to the region of amide stretching vibrations, with two separate and equal spikes at 1623 cm^−1^ and at 1646 cm^−1^, corresponding to the C=0 joined to the cyclohexyl and to the heterocyclic carbonyl stretching vibrations, respectively [20]. The IR spectrum of PM appears to be the sum of the pattern of the individual components. The FTIR spectrum of the CC sample shows broader and less intense signals with respect to raw PZQ’s. Particularly evident is the difference with the starting drug in correspondence of the stretching of the amide groups, which are involved in molecular packing in the crystal form [22,23] the two original sharp peaks were replaced in the coground spectrum with a broader band. In spectrum of Form B, as already noticed [12], the frequency difference in ν(CO) between the stretching of carbonyl groups is lower than those of original PZQ, due to the presence of anti-conformers in Form B, instead of the syn ones of commercial PZQ crystal structure. In fact, Form B exhibits two signals at 1630 and 1639 cm^−1^ due to the symmetric stretching mode of the carbonyl group joined to the cyclohexyl and to that of the heterocyclic carbonyl, respectively. 

### 2.2. Evaluation of the Activated PZQ-Loaded Spray-Congealed Microparticles

To improve the biopharmaceutical properties of PZQ, the activated materials were loaded into hydrophilic microparticles and their characteristics were compared to microparticles containing either raw PZQ or the PZQ:PVP PM. Four batches of MPs (A–D) were then prepared; their composition is reported in Table 2. The real amount of non-degraded PZQ in the cryo-comilled samples was first assayed, in order to prepare the PM and then to calculate the amount of sample to load into the microparticles. After cryo-comilling the 50:50 w/w PZQ:PVP mixture, the effective drug content was about 46% (Table 2).

**Figure 5:**
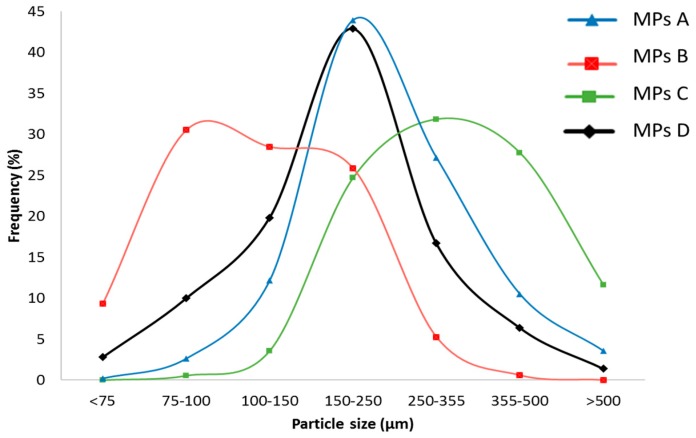
Particle size distribution of the microparticle formulations.

It is worth noting that the raw PZQ, the PZQ:PVP PM, and the PZQ:PVP CC remained suspended in the molten Gelucire^®^ 50/13. Conversely, the addition of PZQ Form B to the molten excipient formed a clear solution, suggesting the formation of a solid solution for MPs B, rather than a solid dispersion, as supposed for MPs A, C, and D, containing cryo-comilled, PM and raw PZQ, respectively. Microparticle size analysis shows that all MPs ranged between 75–500 µm, with different distribution in sizes (Figure 5).

Both MPs A and D displayed a Gaussian curve with the 150–250 µm as a prevalent fraction, while MPs C was found to be bigger, with a broader distribution. MPs B were on average smaller—more than 90% of MPs B had dimensions lower than 250 µm. This different trend was probably due to lower viscosity of the solution, with respect to the suspensions sprayed at the same pressure. In fact, the viscosity of the fluid increased from 100 mPa·s of molten carrier to 960 mPa·s of mixture MPs D, in the following order: MPs B< MPs D<MPs A<MPs C. Therefore, the viscosity of the fluid was not only influenced by the amount of powder included in the molten carrier but also by the particle morphology (size and shape) and by the drug solid state. In fact, the MPs D fluid, containing bigger acicular particles was more viscous than the MPs B fluid, having small dispersed particles and a certain amount of molecularly dispersed drug. MPs A and MPs C fluids differed from the dimensions of the dispersed particles and the viscosity increased proportionally to the particle size. 

**Figure 6:**
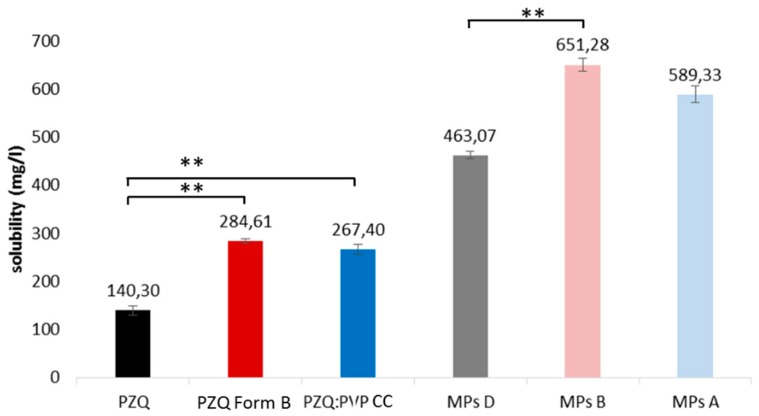
Comparison of the solubility data of the analyzed samples (**indicates significant difference).

All microparticles displayed a drug loading, very close to the theoretical one, with encapsulation efficiencies higher than 90%, for all batches (Table 2). As regards to the solubility tests, the main goal was to verify the effect of the Gelucire^®^-based MPs on the PZQ solubility and, second, to assess the effect of the combination of a mechanochemical activation and spray congealing. The results, reported in Figure 6, clearly demonstrated that the adopted manufacturing and formulation strategies were very useful to enhance the solubility of the BCS class II drug. MPs A increased the drug solubility by four and two times, compared to PZQ and PZQ:PVP CC, respectively. Nevertheless, the more pronounced solubility increase occurred for MPs B, as their solubility was five times greater than the raw PZQ and more than twice that of PZQ Form B. Thus, the solubility of these MPs containing the new polymorph was found to be the highest among the samples. The statistical comparison revealed that solubility of MPs B was significantly higher than that of MPs loaded with PZQ (MPs D), while there were no significant differences among the other MPs.

**Figure 7:**
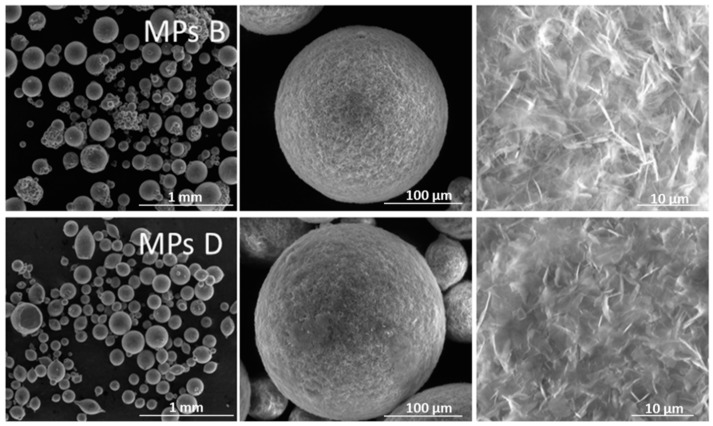
Environmental Scanning Electron Microscopy (ESEM) pictures of MPs B (containing Form B) and MPs D (containing PZQ) (magnification 60×, 500×, and 4.0 K×, in the order from left to right).

In agreement with previous findings [15], the surface of both MPs B and D appeared to be quite rough (Figure 7), which is a typical feature of Gelucire^®^ 50/13-based microparticles obtained by the spray congealing technique. The morphological analysis also revealed that the MPs D sample mainly consists of spherical non-aggregated microparticles, and only a few elongated particles could be detected. On the contrary, the MPs B sample, was characterized by highly spherical particles with polydispersed dimensions and with a remarkable presence of aggregates of very small particles. This was a further confirmation that the addition of micronized particles into the fluid to be atomized, favored the formation of smaller droplets.

The dissolution test was then performed to highlight the difference between the starting samples and the MPs formulations, in the PZQ dissolution rates, and to identify the best formulation approach. Since the dissolution rate varied, depending on the size, wettability, and drug solubility, any factor that might increase one of these properties might have improved the overall bioavailability.

**Figure 8:**
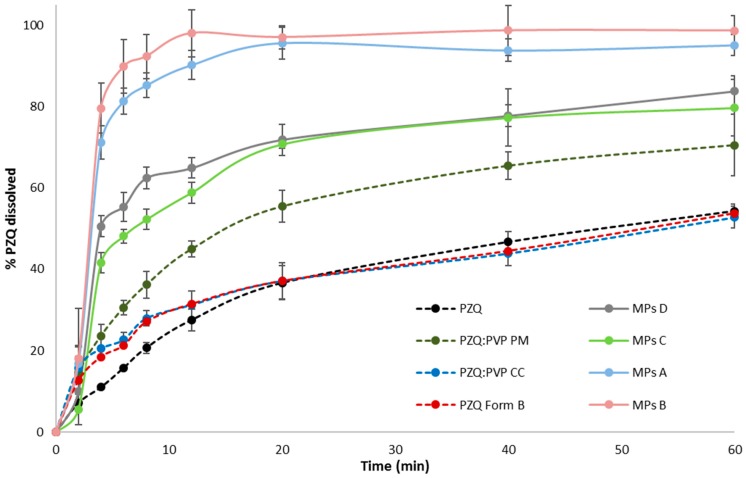
Dissolution profiles of the activated materials and microparticles, in comparison to raw PZQ and a PZQ:PVP physical mixture.

Figure 8 shows the results of the analyzed samples. Analyzing the first 20 min, both ground samples displayed a slight increase of the dissolution rate, relative to the pure PZQ, presumably due to the modification of the crystalline state of the PZQ, both in the CC (semi-crystalline state) and in the milled PZQ (polymorphic form B). In fact, the wettability of Form B meant that it was quite similar to that of the pure PZQ—mean contact angles were 68.77 ± 1.46° and 81.73 ± 1.87°, respectively. However, looking at the whole dissolution profile, the differences were not significant (*f*_2_ = 62.5), in comparison to the pure drug, and the extent of drug dissolved was equal to the raw PZQ (about 52%). This behavior was unexpected, considering their higher solubility, in comparison to the raw PZQ, and it could be explained by taking into account the difference in mean particle size and in particle aggregation—the remarkably higher particle diameter in both ground samples could be a reason behind this dissolution of performance.

Conversely, a significant improvement of the dissolution rate could be obtained from the physical mixture—after 20 min the 55% of the drug was dissolved as compared to 35% of the raw PZQ (*f*_2_ = 42.6), while after 60 min, the amount of drug solubilized was about 66%. Comparing the PZQ:PVP PM with the CC, the difference between the dissolution profiles was borderline significant (*f*_2_ = 49.7). Although the PVP was present in both systems, in the physical mixture, the drug and excipient were simply blended and, thus, did not interact with each other [24]; unlike in the CC system, the milling process led to the formation of bigger aggregates, decreasing the dissolution rate.

Considering the microparticles, all formulations showed a great improvement in drug dissolution rate, compared to the raw PZQ and ground PZQ, confirming that the spray congealing technology enabled the formation of a system with excellent biopharmaceutical properties. Previous paper evidenced that Gelucire 50/13, being a mixture of mono-, di-, and triglycerides, and PEG 1500 esters of stearic acid (HLB 13), it was able to promote micelles formation, consequently, increasing the dissolution rate of Glibencamide [16]. Moreover, it was reported that Gelucire 50/13 microspheres release the entrapped piroxicam via the formation of a lyotropic liquid crystalline phase, which allowed the dissolution of the drug particles in a finely divided, high surface area, and a well-wetted state [19]. In the case of the PZQ-Gelucire 50/13 system (MPs D), the dissolution profiles were significantly different from raw PZQ (*f*_2_ = 24.3), confirming its ability in enhancing the dissolution drug dissolution rate. Hence, although the MPs had bigger particle sizes than raw PZQ, the greater solubility of microparticles D (Figure 5) represented the main driving force in the dissolution rate enhancement. MPs A and B displayed the highest dissolution profiles, without being different from each other (*f*_2_ >50), while being different from MPs D, with *f*_2_ values of 34.6 and 27.2, respectively. In the case of MPs A containing the cryo-coground system, the combination of the drug in the nano-crystalline state and amorphous form, derived from the milling process, with the dispersion into Gelucire 50/13 contributed to the increase of the dissolution performance. For MPs B, in addition to the high solubility of the polymorphic form (Figure 5), the high dissolution rate of the drug could be attributed to the solubilization of Form B, into the molten carrier, during the MP preparation, forming a single-phase system. Thus, the solid state of the activated drug might be changed. 

**Figure 9:**
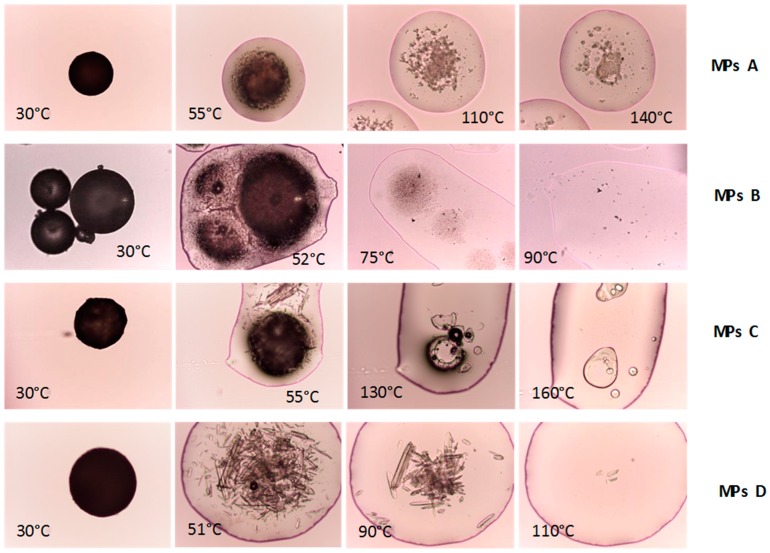
HSM images of microparticle formulations.

In order to better elucidate the drug solid state into the MPs, thermal analysis by means of DSC and HSM were performed. DSC studies of microparticles (graph not shown) revealed the presence of the carrier melting peak in all formulations and the disappearance of the PZQ endotherm, apart from MPs C, which displayed a broad peak at about 130 °C. Since PZQ might have completely solubilized in the molten Gelucire, during the DSC scan, and its melting point might not have been detectable any longer (as observed in previous studies [17]), an additional HSM analysis was carried out (Figure 9). In the case of the PZQ-loaded microparticles (MPs D), at 45 °C, the fusion of Gelucire started at 48 °C and the PZQ acicular crystals were easily recognizable and dispersed in the melted carrier. Afterward, the gradual dissolution of the crystals took place and had completed at about 110 °C, with a clear and transparent appearance of the sample. Therefore, PZQ was still present in the MPs D in the form of acicular crystals, a characteristic that could not be deduced from the DSC thermogram. The disappearance of the drug melting peak in their DSC trace was mainly due to the dissolution of the drug in the melted Gelucire, during the analysis, rather than due to a possible amorphization or change in its solid state, during the spray congealing process. This also meant that PZQ maintained its starting acicular habitus, previously reported in [11,12], even after the spray congealing.

The microscopic analysis of MPs A, containing the cryo-coground, revealed that after the fusion of the Gelucire, the activated system started its melting at 55 °C (black mass reduction), and only at 150 °C, the fusion was completed. At this temperature, the PVP polymer (Tg = 150–180 °C) also appeared in a rubbery state, forming a system with a biphasic appearance, as clearly shown in the picture at 140 °C. These images confirmed the presence of a certain amount of PZQ, in the nanocrystalline form, within the cryo-comacinate (Figure 2). The observation of MPs C, during heating, revealed that after the fusion of Gelucire, both needle PZQ crystals and round PVP particles could be clearly recognized. Additionally, in this case, the disappearance of the PZQ crystals was gradual and started from 50–60 °C, depending on the crystal size and completed at 110 °C, while the PVP completed its phase transition at a higher temperature (160 °C). Finally, the images of MPs B, containing the PZQ polymorphic variety, highlighted the presence of dark spots with a crystal lattice appearance inside the molten droplets, at about 52 °C. Their amount and their dimensions were dramatically reduced, in comparison to what was seen for MPs D, attesting that the residual limited crystalline content of Form B was in the form of a very fine dispersion in the molten Gelucire. Increasing the temperature, they progressively solubilized, until their complete disappearance (at about 90° C, at a temperature lower than the Form B melting point, 112 °C). 

**Figure 10:**
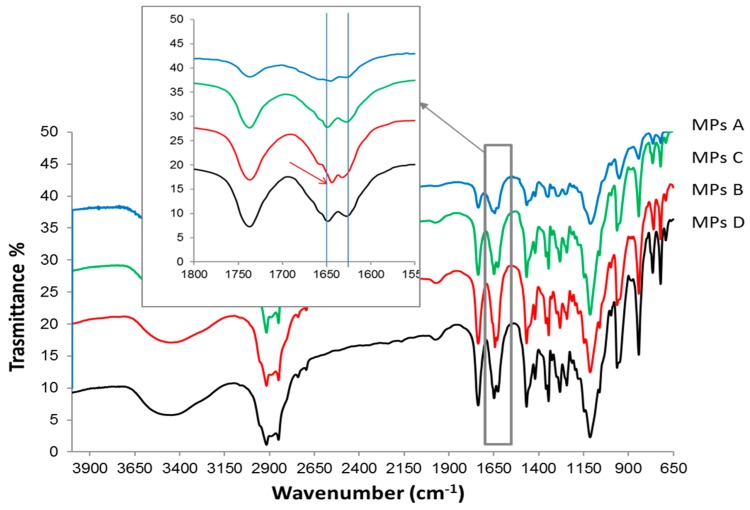
FT-IR spectra of the microparticle formulations.

FTIR spectra of the MPs (Figure 10) were dominated by the Gelucire 50/13 bands, due to its high content in the samples. In particular, in agreement with Brubach et al. [25], particularly evident were the bands in the 1200–1100 cm^−1^ range, while carbonyl stretching vibration appeared at 1715–1738 cm^−1^·s. This permitted the detection of MPs A, MPs C, and MPs D in the two previously mentioned carbonyl stretching vibrations of PZQ, at 1623 cm^−1^ and 1646 cm^−1^, which remained unchanged. On the other side, MPs B also displayed two distinct peaks at 1628 cm^−1^ and 1640 cm^−1^, as is typical of the PZQ Form B [12].

**Figure 11:**
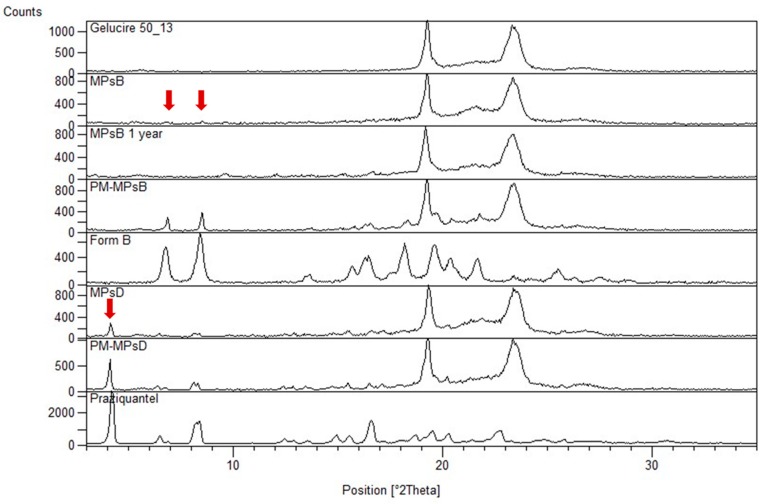
X-Ray Powder Diffraction Studies (PXRD) analysis of Gelucire 50/13, PZQ raw, PZQ Form B, MPs D (red arrow indicates a typical PZQ reflection), and MPs B (fresh samples and after 1 year storage) (red arrows indicate residual Form B signals) and their corresponding PM.

To gather more information about the solid state of PZQ in MPs B, PXRD analysis was performed, and the spectra of both MPs B and D and their corresponding physical mixture (PM) were compared. PXRD pattern of the samples are reported in Figure 11. These results attested that in the microparticle MPs D, the main reflections of the starting PZQ were still visible, confirming the presence of the crystalline drug in this sample. The comparison with the corresponding physical mixture (PM (MPs D)), had an identical composition, revealing that was likely that during the microparticles formation, a slight amorphization or drug dissolution in the Gelucire 50/13 happened, anyway, though PZQ mainly remained in its original crystalline state. Analyzing MPs B and the relative physical mixture, the disappearance of the signal at about 4° of 2θ, in both samples, could be clearly noticed, which is typical of the pure PZQ [11,12]. In addition, the signal attributable to the polymorphic form B was less intense (as evidenced by the arrows), demonstrating the massive molecular dispersion/amorphization of Form B in the carrier. Only a very limited amount of Form B crystals was still present inside the microparticles. The diffractograms of the MPs B showed no evident change in the pattern, compared to the fresh samples, suggesting the stability of the PZQ amorphous form, within the microparticles. Additionally, the dissolution profile of the MPs B remained unchanged, after 1 year of storage, thus, confirming the stability of the pharmaceutical performance of this formulation. 

Finally, the in vitro antischistosomal activity of the compounds was tested on NTS and adult *S. mansoni*. The IC_50_ values on NTS were 3.16 µg/mL for MPs B, 2.4 µg/mL for the Form B and 2.58 µg/mL for the PZQ. On *S. mansoni* adult worms we determined an IC_50s_ of 0.48 µg/mL for MPs B, and slightly lower values of 0.23 µg/mL for the milled PZQ and 0.13 µg/mL for the standard PZQ. Therefore, MPs was found to show a very similar bioactivity to that of PZQ. This meant that the microparticles permitted the release of the active drug and maintenance of the activity against *S. mansoni* adult worms.

## 3. Material and Methods

### 3.1. Materials

Praziquantel (PZQ) Ph. Eur. grade ((11bRS)-2-(Cyclohexylcarbonyl)-1,2,3,6,7,11b-hexahydro-4-H-pyrazino[2,1-a]isoquinolin-4-one) was kindly donated by FATRO S.p.A., Ozzano Emilia, Bologna, Italy. PZQ impurities: Impurity A (2-Benzoyl-1,2,3,6,7,11b-hexahydro-4-H-pyrazino[2,1-a]isoquinolin-4-one) and impurity B (2-Cyclohexanecarbonyl-2,3,6,7-tetrahydro-pyrazino[2,1-a]isoquinolin-4-one) were of Ph. Eur. grade and were purchased from Endotherm Gmbh (Saarbruecken, Germany). Povidone (Kollidon K30, PVP K30) was supplied by BASF (Ludwigshafen, Germany) while Gelucire 50/13 was kindly supplied by Gattefossè (Milan, Italy).

### 3.2. Preparation of Activated Materials by Neat Grinding

These experiments were performed in a vibrational mill-Retsch MM400 (Retsch GmbH, Haan, Germany), which was equipped by two screw-type zirconium oxide jars, each with a capacity of 35 mL. A ceramic material like zirconium oxide was selected, due to its high density (5.9 g/cm^3^). Three zirconium oxide spheres of 15 mm (weighing 10.72 g) were used as the milling media.

In particular, to obtain the PZQ crystalline polymorphic form, Form B, PZQ was ground by itself. The vibrational frequency was set at 20 Hz, for 240 min, without interruption. The amount of powder to be introduced in the milling jar was determined to be 0.800 g per jar, and no cooling was provided to the grinding jar, during room temperature milling. These process parameters were necessary to obtain the new polymorphic form, Form B, which were selected on the basis of our previous work [11]. A 20 Hz vibrational frequency was applied for a duration of 4 h. The experiment was performed, twice, to obtain enough material for both the experimental analysis and the spray congealing process.

For the preparation of the coground system, PZQ and PVP were manually gently mixed in an agate mortar, in a 1:1 drug-to-polymer weight ratio, for the standardized time of 3 min (batch size ranging about 1g). On the basis of previous experiences [11], the amount of powder to be introduced in the milling jar was determined to be 1.072 g per jar, and a vibrational frequency and a milling time of 60 min were set. Prior to milling, the jars containing the samples were immersed in liquid nitrogen for 1 min; re-cooling of the milling jars with liquid nitrogen for 1 min, was performed, every 15 min of milling. The experiment was repeated four times, to obtain enough material for both the experimental analysis and the spray congealing process. 

Post milling, all samples were collected and stored in glass vials in the dark, in a desiccator, over anhydrous calcium chloride, at 25 °C, for further characterization and processing.

For comparison purposes, the properties of the raw PZQ and binary physical mixtures (prepared in the same agate mortar, by manually mixing PZQ and PVP), were investigated.

### 3.3. Preparation of Microparticles by Spray Congealing

Four batches of microparticles (indicated as MPs A–MPs D) using Gelucire^®^ 50/13 as the carrier were produced by the spray-congealing technology, using an external-mix two-fluid atomizer, called Wide Pneumatic Nozzle (WPN) [18]. Gelucire^®^ was heated up to about 10 °C, above its melting point. The active material (whose amount was determined in order to have a final percentage of PZQ equal to 15% *w*/*w*) was added to the molten carrier, as a powder, and magnetically stirred. The suspension or solution obtained was then loaded into the feeding tank of the spray congealing apparatus. The temperature of the feeding tank of the nozzle and the inlet air pressure were set at 60 °C and 3 bar, respectively. The atomized molten droplets hardened during the fall into a cylindrical cooling chamber, which was held at room temperature. Finally, the microparticles were collected from the bottom of the cooling chamber and stored in polyethylene closed bottles, at 25 °C. The batch size was 10 g for each formulation. The composition of the produced microparticles is reported in Table 2.

### 3.4. HPLC Analysis

HPLC analysis was performed for the quantification of both PZQ and the related impurities (or other detectable related products), in the activated samples, after the milling process and for the determination of the real drug content in the microparticles. HPLC analysis was also used to calculate the solubility and the dissolution properties of the different samples. The method was adapted from literature [26] and have been already validated and employed for PZQ quantification in previous studies [11,12,13]. Briefly, the HPLC system consisted of two mobile phase delivery pumps (LC-10ADvp, Shimadzu, Japan) and a UV–vis detector (SPD-10Avp, Shimadzu, Japan). An autosampler (SIL-20A, Shimadzu, Japan) was used to inject samples (20 µL) onto a Kinetex 5 µm C18 column (150 mm × 4.60 mm; Phenomenex, Bologna, Italy). The mobile phase was methanol and water at a ratio of 65:35 V/V, the flow rate and the wavelength of the UV detector were set at 1 mL/min and 220 nm, respectively. The linear calibration curve of the PZQ was obtained in the range of 0.4–40 mg/L (r^2^ = 0.99985). The retention time of PZQ was about 5.5 min and the run time was set at 12 min. The PZQ standard solution was prepared by dissolving 10 mg of PZQ in 20 mL of methanol and diluting 1:200 in the mobile phase, in order to have a final standard PZQ concentration in solution of 2.5 mg/l. The calibration curve of the PZQ specified impurities (impurity A and B according to the PZQ monograph) [27], was obtained in the range of 0.01–1 mg/L (r^2^ = 0.99927 and 0.99941, for impurity A and B, respectively). The retention time of the impurities were at 3.45 min and 11.2 min.

PZQ content was determined by dissolving a variable quantity of the specific sample (PZQ milled, PZQ:PVP cryo-comilled and loaded-microparticles) accurately weighed in 20 mL of methanol. In the case of the milled samples, the obtained solution was then diluted 1:200, in the mobile phase, corresponding to about 2.5 mg/L of PZQ, while for the microparticles, the solution was stirred for 24 h, protected from light, to assure a complete solubility. Finally, the solution was diluted in the mobile phase 1:100. For all samples prior to injection, the solutions were filtered through a 0.2 µm membrane filter and the drug content was assayed by HPLC. Each sample was analyzed in triplicates and the mean of the sum of the peak responses of PZQ was then calculated and have been reported along with the SD. Moreover, for the activated materials, the PZQ recovery was expressed as the percentage of PZQ, with respect to the sum of all peaks (PZQ and related impurities or other detectable related products). However, for the microparticles, the encapsulation efficiency (%) was then calculated by dividing the experimental drug content with the theoretical one and then multiplying it by 100.

### 3.5. Solubility and Dissolution Studies

Solubility and dissolution studies were carried out for both raw and activated materials and for the microparticles. For the solubility test, an excess amount of sample was added to the 10 mL of distilled water. The samples were magnetically stirred for 48 h, at 20 °C, and were protected from light by means of an aluminum foil, throughout the experiment. After equilibrium, the samples were centrifuged at 10,000 rpm, for 20 min, and the supernatant was filtered through a 0.20 mm membrane filter. After diluting the samples in a ratio of 1:200 in the mobile phase, they were finally analyzed by HPLC. The measurements were performed in triplicates, for each formulation, and the mean ± SD was reported. The statistical assessment of the obtained values was performed using one-way ANOVA, while comparison between means was performed using the *t*-Test. Differences were considered statistically significant for *p* values < 0.01.

In vitro dissolution studies of all samples were performed in 1000 mL of water, maintained at 37 ± 0.5 °C, and stirred at 100 rpm, using a paddle apparatus (Erweka DT800, Heusenstamm, Germany). Sink conditions were ensured by considering the PZQ water solubility at 37 °C of 215.0 ± 4.9 mg/L, as found by Trastullo et al. [7], and different amount of samples (corresponding to 16 mg of PZQ) were added to the vessel, according to their composition. Then aliquots of 2 mL were withdrawn at specified times, through a 8 µm filter, in order to only collect the dissolution media and leave the formulation in the vessel. At each sampling time, the PZQ content was assayed by the HPLC. Withdrawn samples were replaced with an equal volume of fresh medium. The dissolution tests were performed, at least in triplicates, and the mean ± SD was reported. Comparison between drug release profiles from the pellets were carried out, using the similarity factor (*f*_2_).

$$f_{2}=50*\log\left\{1+{\left[\frac{1}{n}*\overset{n}{\underset{t=1}{\sum }}{\left(R_{t}-T_{t}\right)}^{2}\right]}^{-0.5}*100\right\}$$

where *n* is the sampling number, *R_t_* and *T_t_* are the cumulative percentage drug dissolved of the reference and the test products at each time point *t*. For the *f*_2_ values calculation, sampling number obtained within 20 min of the dissolution test were considered. The similarity factor fits the result between 0 and 100. The two drug release profiles were similar if the *f*_2_ was greater than or equal to 50.

### 3.6. Wettability Studies

The measurements of contact angle were carried out according to the sessile drop method on compressed non-disintegrating disks, using deionized water as a wetting liquid, as previously reported for Gelucire-based matrices [28]. Disks were prepared by compressing the mixtures in a manual press Perkin Elmer, imparting a force of 1 tons for 1 min. The flat tablets produced were then analyzed with the Drop Shape Analysis System (Krüss DSA 30, Krüss GmbH, Germany), using a single drop of purified water (25 µL). The contact angle (between the disk and the drop) measurements, performed in triplicates, were taken after 10 s. The pure PZQ and milled PZQ were analyzed and the mean of at least three determinations, was calculated.

### 3.7. Viscosity Measurements 

The viscosity of the molten mixtures, heated to the temperature set for the spray congealing process (60 °C), was measured with a Brookflied DVzT viscosimeter (Ametek GmbH, Lorch, Germany) using a spindle number RV04 and a rotating speed of 200 rpm.

### 3.8. Scanning Electron Microscopy (SEM)

Powder samples (pure PZQ, polymorphic form B and cryo-coground) were metallized with S150A Sputter Coater (Edwards High Vacuum, Crawley, West Sussex, UK) and then observed under a scanning electron microscope Leica Stereoscan 430i (Leica Cambridge Ltd., Cambridge, UK). 

### 3.9. Environmental Scanning Electron Microscopy (ESEM)

The shape and surface characteristics of the microspheres were observed by environmental scanning electron microscopy (ESEM) (Quanta 200 FEI, FEI Company, Czech Republic).

### 3.10. Particle Size Analysis 

Particle size measurements of the starting PZQ, Form B, cryo-coground and corresponding PM, were carried out, using a laser diffractometer (Malvern Mastersizer 2000, Malvern, UK). Before analysis, about 10 mg of each sample were dispersed by sonication in 100 mL of water containing 0.1% of polisorbate 80; sample containing PVP were dispersed in silicon oil (Silico DC 245 DOW Corning, Biesterfeld Spezialchemie GmbH, Hamburg, Germany).

Size distribution of microparticles was evaluated by sieve analysis, using a vibrating shaker (Octagon Digital, Endecotts, London UK), and a set of six sieves, ranging from 75 to 500 μm (Scientific Instrument, Milan, Italy).

### 3.11. Differential Scanning Calorimetry (DSC) Studies

Raw materials, activated materials, and MPs were analyzed by DSC, using a Perkin Elmer DSC 6 (Perkin Elmer, Beaconsfield, UK), with nitrogen as the purge gas (20 mL/min). The instrument was calibrated with indium and lead, for temperature, and with indium for the measurements of enthalpy. About 8–9 mg of the sample were placed in an aluminum pan and heated from 30 to 180 °C, at a scanning rate of 10 °C/min. Residual crystallinity (%) was then calculated by the measurements of the enthalpy of fusion (∆H) of the PZQ, using the following equation: Residual crystallinity (%) = (ΔH_sample_ × 100)/ΔH_PZQ_.

### 3.12. Hot Stage Microscopy (HSM) Analysis 

HSM was performed on raw PZQ, PZQ:PVP physical mixture, activated materials and MPs, using a hot stage apparatus (Mettler-Toledo S.p.A., Novate Milanese, Italy), under a Nikon Eclipse E400 optical microscope. Solubilization processes, phase transitions, or polymorphic changes occurred during the heating (from 25 to 200 °C, scanning rate 10 °C/min) were observed and images were captured by means of a Nikon Digital Net Camera DN100. The magnification was set at 10×.

### 3.13. X-Ray Powder Diffraction Studies (PXRD)

PXRD patterns were recorded using a Bruker AXS D5005 X-ray Diffractometer (Karlsruhe, Germany) with Cu-Kα radiation (1.5418 Å), monochromatized by a secondary flat graphite crystal. The analyses were performed in duplicates, using a current of 30 mA and the voltage was set at 40 kV. The powder samples were scanned in the range of 3°–35° of the 2θ angle, steps were of 0.05° of 2θ, and the counting time was of 5 sec/step. The samples subjected to the analysis were the following: Gelucire 50/13, raw PZQ, polymorphic form B, MPs B and MPs D, and the corresponding PMs. 

### 3.14. Fourier Transform-Infrared Spectra (FT-IR) Analysis 

Studies of infrared spectra of the excipients (PVP and Gelucire 50/13), raw PZQ, PZQ:PVP physical mixture, the activated materials, and the MPs were conducted with an IR spectrophotometer (Jasco FT-IR A-200, Pfungstadt, Germany), using the KBr disc method. The samples were mixed with KBr and compressed into a tablet (10 mm in diameter and 1 mm in thickness), using a hydraulic press (Perkin Elmer, Beaconsfield, UK), at 3 tons, for 3 min. The scanning range was 650–4000 cm^−1^ and the resolution was 1 cm^−1^.

### 3.15. Physical Stability During Storage

In order to check possible modifications of the solid state within time, PXRD analyses was done, and dissolution tests of the MPs were carried out after 1 year.

### 3.16. In Vitro Studies on S. Mansoni

Newly transformed Schistosomula (NTS) were obtained using the mechanical transformation method [29] and maintained at 37 °C, 5% CO_2_, for 24 h before the experiments. One hundred NTS were placed in each well of a 96 well plate, containing Medium 199, supplemented with 5% iFCS and 1% penicillin/streptomycin. A concentration of 100 µg/mL PZQ and Form B and MPs B were used and serially diluted 1:3, up to 1.23 µg/mL. The plate was then incubated at 37 °C, 5% CO_2_, and monitored at 24 h, 48 h, and 72 h. The assay was performed with biological triplicates, for each concentration. At each time point the NTS were assessed, microscopically, using a viability scale (3 = motile, no changes to morphology; 2 = reduced motility or some damage to the tegument noted; 1 = severe reduction to motility or damage to the tegument observed; 0 = dead). NTS incubated in the highest concentration of DMSO served as the negative controls.

Adult *S. mansoni* were collected from the hepatic portal and mesenteric veins of the infected mice. In a 24-well plate, 2–3 worm pairs were placed in the culture medium (RPMI supplemented with 5% iFCS and 1% penicillin/streptomycin) with 0.33, 0.11, and 0.037 μg/mL of the test compounds for 24 h, 48 h, and 72 h, at 37 °C, 5% CO2, in biological duplicates. Effects were assessed microscopically, as described above. Adult worms exposed to the maximum concentration of DMSO served as the negative control. IC_50_ values for both NTS and adult worms were calculated using the CompuSyn software (ComboSyn Inc., Paramus, NJ, USA).

## 4. Conclusions

Recent developments of praziquantel formulations that are useful for the preparation of a more appropriate pediatric dosage form, were demonstrated and discussed. The mechanochemical activation, whether in the presence or in the absence of povidone, had resulted in a remarkable increase of the solubility of the starting drug. The milling at room temperature and the cryo-comilling of PZQ, with the polymer, in a 1:1 weight ratio, enabled the improvement of the biopharmaceutical properties, by obtaining either a polymorph (Form B) or an amorphous/nanocrystalline form, with a reduction in crystallinity of about 70%, respectively. The results attested the great potentiality and effectiveness of the method, especially by neat grinding, where the absence of the polymer avoided the dilution of the drug and fulfilled the principle of minimizing the use of excipients in pediatric formulations. However, the unfavorable technological characteristics of the activated systems, such as poor wetting and flowability, caused by electrostatic and cohesive forces, might limit their use in the pharmaceutical production process. On the contrary, Gelucire^®^ 50/13 microspheres, in which the activated systems were encapsulated by means of the spray congealing technology, showed a further increase in solubility and a marked improvement in the rate of dissolution of the drug. After the spray congealing process, the results evidenced that the cryo-coground and the milled PZQ formed either a solid dispersion (nanocrystalline and partial amorphous phase) or a solid solution (completely amorphous state), respectively. As a consequence, the MPs containing both activated systems showed a further increase of the biopharmaceutical properties, compared to the milled powders. The in vitro antischistosomal activity showed that MPs enabled the PZQ release, while maintaining its in vitro activity.

To conclude, the approach consisting in the association of the spray congealing with the mechanochemical activation grinding, in the absence of the polymer, was the most favorable, thus, it is a promising product for designing a new praziquantel formulation and a valid option for enhancing the performance of this antischistosomal drug, possibly permitting a significant reduction in the therapeutic dose.

## Figures and Tables

**Table 1:** Characteristics of the mechanochemically activated materials, in comparison to the raw Praziquantel (PZQ) and PZQ:PVP physical mixture.

Samples	HPLC Assay: Impurity Retention Time (min) (Content, %)			PZQ Recovery (%)	Solubility (mg/L)	Endothermic Peak (°C)	Residual Crystallinity (%)
	Impurity A	Impurity B	Impurity X				
**PZQ**	- *	- *	- *	100	140.30 ± 9.26	143.51 ± 0.35	100
**PZQ-PVP PM**	- *	- *	- *	99.98 ± 0.04	151.78 ± 27.22	142.31 ± 0.22	100
**PZQ-PVP CC**	3.4–3.5 (0.18)	- *	4.0–4.2 (0.93)	98.89 ± 0.08	267.40 ± 11.00	129.36 ± 0.43	27.70
**PZQ Form B**	- *	- *	- *	99.55 ±0.05	284.61 ± 4.67	112.50 ± 0.32	n.a. **

* not present; ** not applicable since a new polymorphic form was obtained.

**Table 2:** List of the analyzed samples, their relative composition and effective drug content.

Samples (Abbreviation)				Composition (%, *w*/*w*)			Real Drug Content (%)	Encapsulation Efficiency (%)
				PZQ	PVP	Gelucire 50/13		
**Powders**	Activated materials	PZQ:PVP cryocomilled	(PZQ:PVP CC)	50	50	-	46.0 ± 1.2	-
		Milled PZQ (Form B)	(PZQ Form B)	100	-		100	-
	Raw materials	PZQ:PVP physical mixture	(PZQ:PVP PM)	50	50	-	46	-
		Raw PZQ	(PZQ)	100	-	-	100	-
**Microparticles**	MPs	PZQ:PVP cryo-comilled	(MPsA)	15	15	70	13.75 ± 0.14	91.7
		Milled PZQ_Form B	(MPsB)	15	-	85	13.54 ± 0.83	90.3
		PZQ:PVP physical mixture	(MPsC)	15	15	70	14.35 ± 0.18	95.7
		Raw PZQ	(MPsD)	15	-	85	15.16 ± 0.11	101.0
